# A mixed methods assessment of an electronic health record-embedded intervention with supportive education and outreach to increase in-hospital opioid use disorder treatment initiation

**DOI:** 10.1002/jhm.70289

**Published:** 2026-02-19

**Authors:** Susan L. Calcaterra, Steven Lockhart, Eric Grimm, Jodi Summers Holtrop, Angela Keniston, Jason A. Hoppe, Jacob A. Lebin, Ingrid A. Binswanger

**Affiliations:** 1 Department of Medicine, Division of Hospital Medicine, University of Colorado, Aurora, Colorado, USA; 2 Department of Medicine, Division of General Internal Medicine, University of Colorado, Aurora, Colorado, USA; 3 Adult and Child Center for Health Outcomes Research and Delivery Service, University of Colorado School of Medicine and Children’s Hospital Colorado, Aurora, Colorado, USA; 4 Department of Family Medicine, University of Colorado, Aurora, Colorado, USA; 5 Department of Emergency Medicine, University of Colorado, Aurora, Colorado, USA; 6 Rocky Mountain Poison and Drug Center, Denver, Colorado, USA; 7 Institute for Health Research, Kaiser Permanente Colorado, Aurora, Colorado, USA; 8 Colorado Permanente Medical Group, Denver, Colorado, USA; 9 Department of Health Systems Science, Kaiser Permanente Bernard J. Tyson School of Medicine, Pasadena, California, USA

## Abstract

**Background:**

In-hospital opioid use disorder medication (MOUD) initiation remains low despite its effectiveness to reduce mortality.

**Objective:**

Assess whether a non-interruptive electronic health record (EHR)-embedded intervention with supportive education and outreach increased in-hospital MOUD initiation without disrupting clinical workflow.

**Methods:**

In this mixed-methods study across 12 hospitals, we conducted an interrupted time series analysis of hospitalizations involving OUD and/or opioid poisoning. We condcuted a directed content analysis of interviews from 60 hospital-based clinicians, social workers, nurses, and pharmacists. The intervention involved MOUD initiation orders, discharge naloxone orders, and access to local OUD treatment resources. Strategies for adoption included clinican and nurse OUD education and required nurse trainings for Clinical Opiate Withdrawal Scale (COWS) assessments. Outcome measures included monthly changes of inpatient methadone administration ≥30 mg, discharge buprenorphine and naloxone prescriptions, and COWS assessments. Emergent themes from qualitative data provided context and possible explanations for quantitative results.

**Results:**

From January 2021 to September 2024, there were 450,790 hospitalizations; 3.1% (*n* = 13,902) involved OUD/opioid poisoning; and, of these, 19.8% (*n* = 2750) involved MOUD. Between the pre- and post-intervention periods, there was no significant change in monthly methadone administrations or discharge naloxone prescriptions. Monthly buprenorphine discharge prescriptions decreased from 10.5% to 8.2% (slope decrease: −0.35, *p* < .001) in the post-implementation period. In the immediate post-intervention period, monthly COWS documentation increased from 7.3% to 14.5% (level change: 6.87; *p* < .001). Qualitative findings reflected a lack of intervention awareness due to limited leadership communication across hospitals.

**Conclusion:**

A non-interruptive EHR-embedded intervention with supportive education and outreach was insufficient to increase MOUD initiation.

## INTRODUCTION

From 2005 to 2021, US opioid-related hospitalizations nearly doubled.^1^ Hospitalization provides an opportunity to diagnose opioid use disorder (OUD) and initiate treatment.^2^ Current hospital-based recommendations include conducting an opioid withdrawal assessment using the Clinical Opiate Withdrawal Scale (COWS),^3^ initiating medications for OUD (MOUD) such as buprenorphine or methadone, providing naloxone at discharge, and ensuring linkage to ongoing OUD treatment.^4,5^ Yet, many adults are not offered MOUD following an acute care encounter related to an overdose^6^ unless they receive care in settings with structured support, such as emergency department (ED) with buprenorphine initiation practices and protocols,^7–9^ or a hospital-based addiction consultation service (ACS).^10–13^

Efforts to increase MOUD initiation and discharge naloxone in EDs and hospitals often rely on electronic health record (EHR)-embedded clinical decision support (CDS) tools. User-centered CDS in EDs has demonstrated mixed effects for buprenorphine initiation and linkage to outpatient OUD care,^7–9^ while CDS interventions to increase naloxone prescribing has been more consistently effective in EDs and hospitals.^14–16^ In hospitals, most strategies to increase MOUD initiation involve an ACS, which support MOUD initiation and broader substance use disorder treatment.^12,17–20^ However, disparities in OUD treatment persist; rural hospitals are 25% less to have an ACS and 50% less likely to support in-hospital MOUD initiation.^21^

To improve in-hospital OUD treatment initiation, we developed an OUD intervention that integrates OUD diagnostic tools, COWS assessment calculators, protocolized methadone and buprenorphine initiation orders, precipitated opioid withdrawal management strategies, naloxone discharge orders, and OUD outpatient resources into a single, systemwide, non-interruptive EHR-embedded CDS tool. Our non-interruptive CDS tool offered clinical guidance without disrupting workflow and was supplemented with supportive education and outreach across a multi-hospital system.^22^ We evaluated its impact by examining changes in methadone administration, discharge buprenorphine and discharge naloxone prescribing, and COWS documentation. To contextualize these results, we conducted interviews with nurses, clinicians, pharmacists, and social workers at the mid-point and post-intervention implementation periods to understand their perceptions of the intervention.

## METHODS

### Study setting and design

The study occurred in Colorado during peak overdose mortality^23^ and periods of high COVID-19 hospitalizations. The health system includes 12 hospitals that share a single EHR and average 174,000 hospitalizations annually.^24^ Across the system, approximately 220 physician hospitalists, 80 advance practice professional (APP) hospitalists, and 2,650 nurses provide inpatient care. The largest hospital, a 775-bed university-affiliated hospital, employs approximately 140 physicians, 60 APP hospitalists, and has an ACS; all remaining hospitals do not have an ACS. In 2019, the system implemented an EHR-based CDS tool to increase naloxone prescribing at discharge for patients at increased overdose risk, independent of this study.^14^

This mixed-methods study used quantitative and qualitative data to evaluate the impact of an EHR-embedded OUD intervention with supportive education and outreach on hospital-based MOUD initiation. Using an explanatory sequential study design,^25^ we used qualitative data to provide possible explanations for the quantitative results.^26,27^ The study was approved by the Colorado Multiple Institutional Review Board.

### The EHR-Embedded OUD intervention

The non-interruptive, EHR-embedded OUD intervention (henceforth “intervention”) was developed by an addiction medicine-trained hospitalist, a social worker, a pharmacist, and a nursing leader, with system-level analyst support. The intervention includes Diagnostic and Statistical Manual of Mental Illnesses 5th Edition diagnostic criteria,^28^ opioid withdrawal assessment using an online COWS calculator,^29^ COWS orders, methadone initiation orders (30–40 mg starting dose up to 60 mg on Day 1 followed by patient-reported symptomatic dose increases), buprenorphine initiation orders (4–8 mg starting dose up to 24 mg on Day 1, followed by patient-reported symptomatic dose increases), discharge naloxone orders, a protocol for precipitated opioid withdrawal management, and links to local outpatient OUD treatment across the state. As a non-interruptive CDS tool, it does not generate alerts; it resides in a separate EHR activity tab and must be accessed by staff who are aware of it. Use of the intervention and its recommendations is optional. Similar non-interruptive interventions are used in the health system to guide care for common conditions, for example, pneumonia and hyponatremia. The intervention was implemented systemwide on January 18, 2023.

### Supportive educative and outreach for intervention adoption

Prior to the intervention’s deployment, the addiction medicine physician delivered 10 voluntary 1-h educational sessions for hospitalists, APPs, and nurses covering the topics of universal screening for unhealthy substance use, COWS assessments (nurses), evidence for MOUD, and “how to” medication initiation recommendations (physicians/APPs), and “how to” access the intervention. Five sessions were in-person, five were virtual and recordings were posted on a system intranet. Sessions occurred from November 2022 to January 2023, and participation was not tracked. Email communications about the sessions were distributed by each hospitalist groups’ leader, inpatient nursing educators, and inpatient pharmacy leaders to their respective staff with two reminder emails. The Chief Nursing Officer also mandated online training modules on “how to” conduct COWS assessments and universal screening (Supporting Information S1: Appendix S1).

### Study population

We examined two populations: (1) patients with ≥1 hospitalizations documenting OUD^30,31^ and/or opioid poisoning^8,32,33^ and (2) hospital-based nurses, clinicians, pharmacists, and social workers.

For the former (hospitalizations involving patients with OUD/opioid poisoning), the unit of analysis was the hospitalization. We identified all hospitalizations across the 12 hospitals from January 1, 2021 to September 30, 2024, and excluded hospitalizations that were <24 h, patients <18 years old, and hospitalizations involving patients with past 30-day use of buprenorphine (milligram and microgram formulations) or methadone for pain or OUD documented at the time of admission. Opioid prescription data were obtained from pharmacy claims, the health system data warehouse, and admission medication reconciliation.

For the latter (hospital staff), we utilizing a convenience sampling strategy and invited hospitalist physicians and APPs, nurses, pharmacists, and social workers via email to participate in key informant interviews or focus groups. We invited people who were familiar with the intervention in order to learn about their experiences interacting with the intervention. We did not track refusals.

Focus groups and interviews occurred 4–7 months after the intervention’s implementation (May–August 2023) and again at 14–18 months after the intervention’s implementation (March–August 2024). Participants unable to attend a focus group were offered an interview. We conducted five focus groups (≈8 participants each) and 19 key informant interviews. A trained qualitative analyst (S.L.) conducted all 1-h sessions, which were recorded via videoconferencing. Participants received a gift card.

### Data sources

We queried routine clinical EHR data, encounters, diagnoses, and medications, from the health system’s data warehouse.^34^ International Disease Classification Codes, 10th Edition (ICD-10) identified encounter-level documentation of substance use disorders (SUD), mental health diagnoses, skin and soft tissue infections (SSTI), endocarditis, and musculoskeletal infections (Supporting Information S1: Appendix S2). We captured in-hospital methadone administration (dichotomized as ≥30 vs. <30 mg/day to capture doses commonly used for OUD vs. pain),^35^ COWS scores,^36^ discharge buprenorphine and naloxone prescriptions, and hospital length of stay.

Demographic data (sex, age, race, ethnicity, insurance status) were derived from each patient’s last hospitalization during the study period, defined as the “index hospitalization.” ICD-10 codes documenting mental health conditions, SUD, and SSTI, endocarditis, and musculoskeletal infections were identified from encounters in the 3 years preceding the index hospitalization (Supporting Information S1: Appendix S2).^37^ We also identified ED visits and hospitalizations in the year before the index hospitalization’s discharge date.

At midpoint and at study end, we conducted key informant interviews and focus groups with hospital staff. Interview guides explored experiences using the intervention, perceived leadership support and promotion to use the intervention, perceived effectiveness of the intervention, and confidence in its use. Study end interview guides also explored strengths and weaknesses of both the implementation approach and the intervention itself.^38,39^ We followed the Consolidated Criteria for Reporting Qualitative Research guideline throughout the qualitative study.^40^

### Outcomes

Co-primary outcomes were changes in monthly percentage of inpatient methadone administrations ≥30 mg (number of monthly inpatient methadone administrations/number of monthly hospitalizations with an OUD and/or opioid poisoning ICD code) and buprenorphine discharge prescriptions (number of monthly discharge buprenorphine prescriptions/number of monthly hospitalizations with an OUD and/or opioid poisoning ICD code). Additional outcomes included changes in monthly percentage of naloxone discharge prescriptions and changes in monthly percentage of COWS documentation. Qualitative themes contextualized and helped explain quantitative results.

### Interrupted time series (ITS) analysis

We calculated monthly percentages of hospitalizations with OUD and/or opioid poisoning and each outcome. We divided data into pre-intervention (January 1, 2021 to December 31, 2022; 24 time points) and post-intervention (April 1, 2023 to September 30, 2024; 18 time points). To measure the effects of the intervention on study outcomes, we conducted an ITS analysis in which we fitted linear segmented regression models assessing for level changes, that is, immediate changes in the fitted line, or slope changes, that is, changes in trajectory of the fitted line over time.^41^ The counterfactual for the level and slope change was based on the fitted line from the pre-intervention period.^42^ For each outcome, we calculated the difference between the observed outcomes in the post-intervention period from the predicted outcomes had the intervention not been implemented. To control for monthly variation unrelated to the intervention such as seasonal variation in hospitalizations and clinical practice variation among hospital staff, all segmented regression models included autoregressive terms. We conducted regional (north, central, south) subgroup analyses to assess for practice variation across the state.

### Qualitative analysis

We analyzed qualitative data using an iterative, team-based directed content analysis until reaching thematic saturation. The study team (S.L., S.L.C) deductively and inductively developed a code book informed by our prior work,^4,43–46^ relevant literature,^2,11,47–50^ and interview guides. We expanded the code book using open coding with debriefing until reaching consensus and strong code assignment agreement. We independently coded transcripts (S.L., S.L.C.) with over 20% double coded, and later merged codes to reach immersion. The study team met regularly to review emerging codes and themes until consensus was reached. ATLAS.ti version 24.0 supported data management.

### Data integration

We collected data using an explanatory sequential design^25^ with quantitative analysis preceding qualitative analysis to contextualize and inform quantitative findings.^51^ The quantitative findings and qualitative data were initially integrated by drafting interview questions and deductive codes based on preliminary quantitative results. We built a joint display with a side-by-side comparison of the two datasets to assess for “fit” of the quantitative and qualitative findings by identifying one of three outcomes between the two data sets: confirmation (findings reinforce each other), expansion (findings diverge and expand insights by addressing difference aspects of the phenomena), or discordance (findings are contradictory).^52^ Finally, we merged the final quantitative results with the qualitative results to further interpret study outcomes.^39,51,53,54^

## RESULTS

### Quantitative results

From January 1, 2021 to September 30, 2024, there were 450,790 hospitalizations involving 275,264 unique patients across 12 hospitals. Three percent (13,902) of hospitalizations, involving 7339 unique patients, met inclusion criteria. The majority (80.2%, *n* = 11,152) of included hospitalizations did not involve in-hospital administration of ≥30 mg of methadone or a discharge buprenorphine prescription despite having OUD/opioid poisoning listed as an encounter diagnosis (Table 1).

Individual patients with OUD/opioid poisoning had higher percentages of Medicaid (54.5% vs. 20.1%), 3-year ICD-10 code documenting anxiety or depression (37.5% vs. 23.2%), psychostimulant use disorder (13.2% vs. 1.1%), alcohol use disorder (12.6% vs. 5.2%), and cocaine use disorder (3.7% vs. 0.4%), and a higher number of past year ED visits (2.0 vs. 1.0) and hospital admissions (0.9 vs. 0.5) compared to patients without OUD/opioid poisoning (Table 2).

#### Changes in study outcomes over time (Slope Change)

**FIGURE 1:**
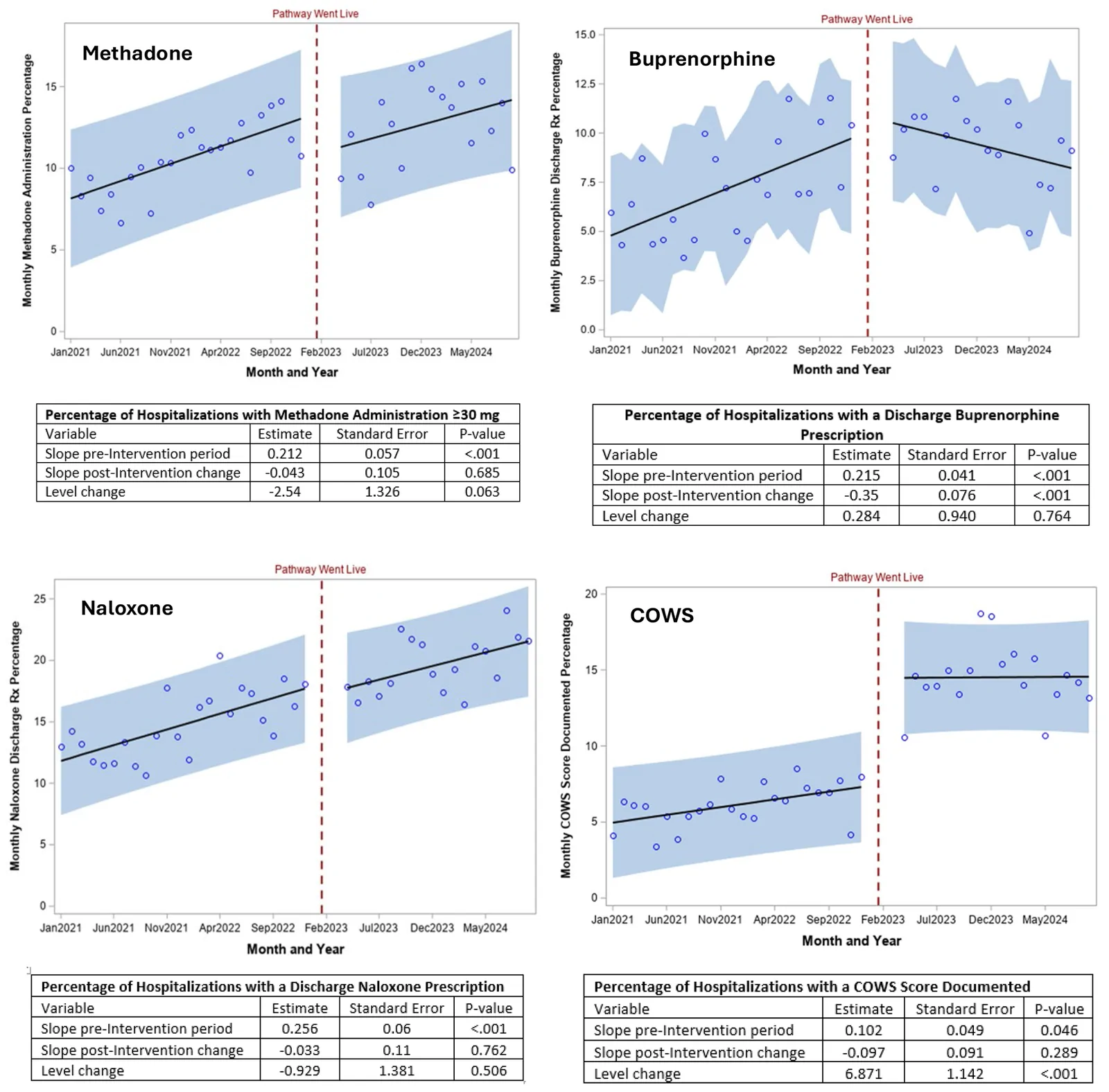
Results of the interrupted time series (ITS) analysis demonstrating the percentage of hospitalizations with an outcome of interest (percentage of: monthly administrations of methadone, buprenorphine discharge prescriptions, naloxone discharge prescription, and Clinical Opiate Withdrawal Scale [COWS] documentation) among patients with documented opioid use disorder (OUD) and/or opioid poisoning pre- and post-implementation of the EHR-embedded OUD intervention.

Compared to the 24-month pre-intervention period, in the 18-month post-intervention period, there was no significant change in the monthly percentage of methadone administrations of ≥30 mg (*p* = .68), the monthly percentage of discharge buprenorphine prescriptions decreased from 10.5% to 8.2% (slope decrease: −0.35, *p* < .001), there was no significant change in the monthly percentage of discharge naloxone prescriptions (*p* = .76), and there was no significant change in the monthly percentage of COWS documentation (*p* =.29) (Figure 1).

#### Immediate outcome changes post intervention

Following the 4-month intervention deployment period, there was a significant increase in the percentage of monthly COWS score documentation in the immediate post-intervention period, that is, level change, increasing from 6.5% in December 2022 to 14.9% in April 2023 (level change: 6.87; *p* < .001) (Figure 1). There were no other significant level changes in the immediate post-intervention period (methadone, *p* = .06; buprenorphine, *p* = .76; naloxone, *p* =.51) (Figure 1).

Similar trends persisted in the immediate and sustained post-intervention periods in the regional analyses for all study outcomes (Supporting Information S1: Appendix S3 and S4).

### Qualitative results

There were 60 key informants or focus group participants representing all three hospital regions: 21 clinicians, 17 nurses, 12 social workers, and 6 pharmacists; 4 participants did not provide demographic information (Table 2).

### Emergent themes

#### **Theme 1:** Limited experience and perceived impact of the intervention, due to the lack of awareness and exposure.

Despite efforts to promote the intervention, many participants reported minimal to no experience using it in clinical practice. While some assumed the intervention improved in-hospital OUD treatment, many were unable to comment due to only hearing about the invention once and never using in practice. Those familiar with the intervention described it as a useful tool to improve their confidence in methadone and buprenorphine initiation, to standardize patient care to reduce patient-directed discharge, and to teach residents or other learners about in-hospital OUD treatment.

#### **Theme 2:** Lack of confidence and comfort of clinicians to initiate MOUD.

While most participants perceived an increase in MOUD initiation, some participants reported a reluctance to initiate methadone due to its complicated pharmacokinetics or avoided buprenorphine out of concern for precipitating opioid withdrawal. Some participants reported a reluctance to initiate MOUD due to unclear post-discharge follow-up plans, e.g., some perceived starting a new medication in the hospital without a discharge plan as futile. Most participants reflected on the need for continued education, training, and support from the health system to increase use of the intervention to facilitate MOUD prescribing.

#### **Theme 3:** A nursing leadership-required training facilitated familiarity with and use of COWS, while improving care for patients with OUD.

Almost all participants noted an uptake in COWS documentation, with most attributing to a nursing leadership required COWS training. Some perceived that clinicians were more likely to address opioid withdrawal treatment if COWS scores were documented in the patient’s chart. This was perceived as a significant benefit when supporting patients experiencing opioid withdrawal. Some participants reflected on the challenges of accurately scoring COWS and determining when to start and stop COWS scoring. The main concerns from those frequently documenting COWS was a perceived lack of clinician initiation of MOUD in response to the COWS score.

#### **Theme 4:** Limited exposure to messaging and endorsement from system leadership about the intervention.

Most participants reported limited or no messaging, endorsement, or education (outside of nursing leadership required COWS training) from system leadership about the intervention. Some vaguely remembered receiving an email about the intervention, while others noted hearing about the intervention from peers or through the email invitation to participate in the study’s interviews. Most participants reported that more could have been done to increase utilization of the intervention and described wanting more education and outreach from system leadership to improve the intervention’s impact. Many described a lack of cohesion and communication across the hospital regions, which may have contributed to disparate OUD treatment provided across the health system’s hospitals.

Table 3 includes emergent themes with representative quotations.

### Mixed methods results

Qualitative findings largely confirmed and expanded on the quantitative results adding detailed explanations to quantitative results. Mixed methods integrated findings are reported in Table 4.

## DISCUSSION

In this mixed-methods multisite study, the EHR-embedded OUD intervention with supportive education and outreach was not associated with increases in-hospital methadone administrations, discharge buprenorphine prescriptions, or discharge naloxone prescriptions. There was an increase in COWS documentation in the immediate post-intervention period. Qualitative results indicated that a lack of awareness of the intervention and limited leadership messaging encouraging use of the intervention, combined with clinician discomfort prescribing MOUD, likely contributed to low adoption of MOUD.

COWS documentation significantly increased in the immediate post-intervention period. The qualitative results suggest that this change may be attributed to nursing leadership’s collaboration with the OUD working group to develop a required nursing online training module for COWS scoring and documentation. Similar findings were not observed with MOUD initiation, whose education was optional. These finding suggests that future in-hospital OUD interventions should involve collaboration between health system leadership and clinicians to encourage practice change, possibly by incentivizing or requiring clinicians to complete OUD education and training, with ongoing clinician incentives to incorporate MOUD initiation into practice.^55,56^ Regulatory bodies, including the Centers for Medicare and Medicaid Services, could monitor and reimburse hospitals using quality of care metrics for OUD treatment. Combined efforts by health system leaders, clinicians, and regulatory bodies may impact MOUD initiation in the hospital setting.^57^

Pre-intervention period trends for in-hospital methadone administration, buprenorphine discharge prescriptions, naloxone discharge prescriptions, and COWS documentation were generally increasing. Most trends continued to increase or remained stable in the post-intervention period. The exception was buprenorphine where the percentage of monthly buprenorphine discharge prescriptions significantly decreased in the post-intervention period. Some participants expressed concern about precipitating opioid withdrawal with buprenorphine which may account for this finding. With fentanyl’s emergence in the drug supply, reports of precipitated opioid withdrawal increased following buprenorphine initiation.^58^ Messaging that buprenorphine remains a highly effective MOUD and should be offered to hospitalized patients with OUD could have improved outcomes.

### Limitations

The quantitative results are observational and may be influenced by unmeasured confounders. Although we excluded encounters involving pre-hospital MOUD, misclassification is possible and could bias results. We also did not capture discharge disposition, including in-hospital death. Qualitative findings may be affected by selection, recall, and social desirability biases, particularly given that the study PI (SLC) directs the ACS at the University Hospital and participated in coding. We did not directly measure fidelity to the intervention or systematically track participation in education and training sessions; instead, we relied on participant-reported awareness and use, an indirect indicator of fidelity.

## CONCLUSIONS

Findings suggest that a non-interruptive EHR-embedded intervention with supportive strategies alone is insufficient to increase hospital-based MOUD initiation. Stronger leadership engagement, along with incentivized education and training, may be necessary to drive meaningful practice change.

## Supplementary Material

Appendix 1

Additional supporting information can be found online in the Supporting Information section at the end of this article.

## Figures and Tables

**TABLE 1:** Encounter level characteristics of hospitalized patients (*N* = 450,790),^a,b^

	Documented OUD or opioid poisoning during one or more hospitalizations in the study period			
	Buprenorphine prescribed at hospital discharge^c^ (*n* = 1131)	Methadone administered with a daily dose ≥30 mg/day^d^ (*n* = 1619)	No methadone administered and no buprenorphine prescribed at hospital discharge^e^ (*n* = 11,152)	All remaining encounters (*n* = 436,888)
Hospital region in the state,^f^ *n* (%)				
North	230 (20.3%)	212 (13.1%)	3474 (31.2%)	138,396 (31.7%)
Central	619 (54.7%)	1063 (65.7%)	3806 (34.1%)	167,442 (38.3%)
South	282 (24.9%)	344 (21.2%)	3872 (34.7%)	131,050 (30%)

Hospital service category, *n* (%)				
Medicine	855 (75.6%)	1112 (68.7%)	8981 (80.5%)	257,807 (59%)
Neurology	5 (0.4%)	11 (0.7%)	39 (0.3%)	4813 (1.1%)
OB-GYN	69 (6.1%)	138 (8.5%)	235 (2.1%)	64,610 (14.8%)
Psychiatry	0 (0%)	0 (0%)	3 (0.03%)	22 (0.01%)
Radiology	0 (0%)	0 (0%)	4 (0.04%)	67 (0.02%)
Physical Medicine/Rehabilitation	21 (1.9%)	27 (1.7%)	86 (0.8%)	6759 (1.5%)
Surgery	178 (15.7%)	330 (20.4%)	1791 (16.1%)	102,321 (23.4%)
Unassigned/Unspecified	3 (0.3%)	1 (0.1%)	13 (0.1%)	489 (0.1%)

Documented diagnosis during hospitalization, *n* (%)				
Schizophrenia	62 (5.5%)	69 (4.3%)	408 (3.7%)	5596 (1.3%)
Bipolar disorder	131 (11.6%)	198 (12.2%)	812 (7.3%)	12,481 (2.9%)
Anxiety or depression	624 (55.2%)	785 (48.5%)	5071 (45.5%)	102,164 (23.4%)
Psychostimulant use disorder	263 (23.3%)	407 (25.1%)	1144 (10.3%)	6237 (1.4%)
Cocaine use disorder	61 (5.4%)	67 (4.1%)	262 (2.3%)	1765 (0.4%)
OUD or opioid poisoning	703 (62.2%)	1134 (70%)	11,152 (100%)	0 (0%)
Alcohol use disorder	229 (20.2%)	175 (10.8%)	1354 (12.1%)	32,722 (7.5%)
SSTI, endocarditis, musculoskeletal infection^g^	137 (12.1%)	299 (18.5%)	1178 (10.6%)	20,817 (4.8%)

Other variables				
Naloxone prescription at discharge, *n* (%)	523 (46.2%)	521 (32.2%)	1383 (12.4%)	8843 (2%)
Length of stay, mean (SD)	9.0 (16.7)	10.1 (19.2)	7.8 (12.5)	4.7 (7.3)
Average first COWS score mean (SD)	7.2 (5.9), *n* = 333 (29.5%)	7.2 (5.5), *n* = 479 (29.6%)	6.9 (5.9), *n* = 621 (5.6%)	6.3 (5.1), *n* = 526 (0.1%)
Average daily methadone dose during hospitalization (mg/day)	49.8, *n* = 82 (7.3%)	74.9, *n* = 1619 (100%)	13.2, *n* = 262 (2.3%)	11.8, *n* = 606 (0.1%)

a The total number of unique hospitalizations is slightly smaller than adding up all four columns because some patients received both buprenorphine and methadone. Those hospitalizations were included in both columns.

b Inclusion criteria was defined as ≥1 hospitalization during the study period with documented opioid use disorder (OUD) or opioid poisoning. Some patients had more than one hospitalization during the study period in which OUD/opioid poisoning was not documented which is why the column does not equal 100%.

c Buprenorphine *was prescribed* at hospital discharge among hospitalizations with a documented OUD or opioid poisoning.

d Methadone *was administered* at a daily dose of ≥30 mg/day among hospitalizations with a documented OUD or opioid poisoning.

e Buprenorphine *was not prescribed* at hospital discharge and methadone *was not administered* at a daily dose of ≥30 mg/day among hospitalizations with a documented OUD or opioid poisoning.

f The three northern hospitals included a 263-bed hospital, a 191-bed hospital, and a 91-bed hospital, the five central hospitals included a 775-bed hospital (which has an addiction consultation services Monday through Saturday), a 93-bed hospital, 83-bed hospital, and a 34-bed hospital, and the four southern hospitals included a 387-bed hospital, a 140-bed hospital, a 13-bed hospital, and a 22-bed hospital.

g Skin and soft tissue infections (SSTI).

**TABLE 2:** Demographic characteristics of patients at the index hospitalization (*n* = 282,603) and key informant/focus group participants (*n* = 60).

	Patients^a^		
	OUD or opioid poisoning (*n* = 7339)	All patients without OUD or opioid poisoning (*n* = 275,264)	Key informant/Focus group participants^2,3^ (*n* = 60)
Sex, *n* (%)			
Female	3818 (51%)	161,408 (58.6%)	43 (70.9%)
Male	3521 (49%)	113,852 (41.4%)	13 (21.3%)
Unknown	0 (0%)	4 (0%)	5 (8.2%)
Age in years, mean (SD)	48.3 (15.5)	55.1 (20.7)	

Age group in years, *n* (%)			
18–30	903 (14.1%)	43,961 (16%)	
31–40	1345 (24.9%)	44,095 (16%)	
41–50	1126 (18.4%)	26,626 (9.7%)	
51–60	1236 (16.6%)	34,111 (12.4%)	
61+	2729 (26%)	126,471 (45.9%)	

Race, *n* (%)			
Asian/Pacific Islander/Native American	188 (2.8%)	9740 (3.5%)	2 (3.3%)
Black/African American	524 (7.2%)	17,761 (6.5%)	1 (1.6%)
White/Caucasian	5622 (74.5%)	209,435 (76.1%)	38 (62.3%)
Other/More Than One Race/Unknown	1005 (15.5%)	38,328 (13.9%)	20 (32.8%)

Hispanic ethnicity, *n* (%)			
Yes	1202 (18.9%)	47,091 (17.1%)	
No	6028 (79.6%)	223,843 (81.3%)	
Unknown	109 (1.5%)	4330 (1.6%)	

Job description, *n* (%)			
Physician or advanced practice provider			24 (39.3%)
Nurse			19 (31.2%)
Social worker			12 (20%)
Pharmacist			6 (9.8%)

Insurance category, *n* (%)			
Medicaid	3065 (54.5%)	55,256 (20.1%)	
Medicare	2749 (27.7%)	109,826 (39.9%)	
Private	986 (11%)	83,272 (30.3%)	
Uninsured/self-pay/indigent/tricare/other/unknown	539 (6.8%)	26,910 (9.8%)	

Documented diagnosis in past 3 years, *n* (%)			
Schizophrenia	315 (5.2%)	3444 (1.3%)	
Bipolar	459 (7.7%)	6134 (2.2%)	
Anxiety or depression	2794 (37.5%)	63,982 (23.2%)	
Psychostimulant use disorder	674 (13.2%)	3120 (1.1%)	
OUD or opioid poisoning	2493 (39.6%)	6147 (2.2%)	
Alcohol use disorder	795 (12.6%)	14,331 (5.2%)	
Cocaine use disorder	168 (3.7%)	1035 (0.4%)	
SSTI, endocarditis, musculoskeletal infection^d^	708 (10.5%)	11,186 (4.1%)	

Acute care utilization, mean (SD)			
Past year ED visits	2.0 (3.6)	1.0 (2.3)	
Past year hospitalizations	0.9 (1.8)	0.5 (1.2)	

a The total number of unique patients with opioid use disorder (OUD) and/or opioid poisoning is slightly smaller than adding up the buprenorphine, methadone, and neither columns since some patients received both buprenorphine and methadone. Those patients were included in both columns.

b There were 60 key informants or focus group participants: 21 clinicians, 17 nurses, 12 social workers, and 6 pharmacists, and 4 participants did not complete demographic data.

c Blank cells mark uncollected data.

d Skin and soft tissue infections (SSTI).

**TABLE 3:** Themes and illustrative quotations organized by topic.

Topic	Theme	Illustrative quote
Experience with the intervention	Limited experience and perceived impact of the electronic health record (EHR)-embedded intervention, due to the lack of awareness and exposure	• “…Unfortunately, I don’t see the OUD intervention used very often in my inpatient population… Most of the time it’s on our recommendations… Then we just have to remind them like, “Hey, we also should be using these protocols [laughter] for their opioid abuse.”” (pharmacist focus group, participant 1) • “I had no clue. That’s why I was so interested in this group because—[laughter] I mean, this is the first I have heard about it.” (social work focus group, participant 1) • “They’re integrated within the EHR, which makes them easy to find. You can click and order things from them.” (hospitalist focus group, participant 6) • “I had my own patient recently and ended up goin’ down the suboxone pathway [embedded in the OUD intervention] on a night shift and got to where I needed to get with the medication, the dosing, really, really pretty quickly. I saw the guy walkin’ around the unit later, and he looked like a completely different guy from admission. It worked like a charm.” (hospitalist focus group, participant 3)
Barriers to initiate methadone or buprenorphine	Lack of confidence and comfort of clinicians to Initiate opioid use disorder medication (MOUD)	• “I think that’s one of the big hesitations of providers not being as familiar with these medicines. If you prescribe them what happens if it precipitates withdrawal?” (physician key informant, participant 7) • “When I was trained long ago, it was like “don’t mess with methadone”…that’s still part of my ethos and methadone scares me. I don’t know if I feel comfortable doing it because it’s such a weird pharmacokinetic medication. Even with the pathway, I don’t know if I feel comfortable.” (hospitalist focus group, participant 6) • “Well, the doctors just don’t know enough about Suboxone to actually prescribe…I think the doctors really need more formal training.” (social worker focus group, participant 2). • “No one’s dedicated to that role in particular. Our social workers are sort of assigned by units. They won’t schedule an appointment for a patient in a Suboxone clinic or anything like that. They’ll provide a list of Suboxone clinics in the community. It’s incumbent on the patient to make that connection.” (physician key informant, participant 5)
Experience with Clinical Opiate Withdrawal Scale (COWS)	A nursing leadership-mandated training facilitated familiarity with and use of COWS, while improving care for patients with OUD	• “…when the intervention first rolled out there was a universal—you learned that every nurse in the system had to do COWS score documentation…” (pharmacist focus group, participant 3) • “…I would say I think when it rolled out, we got decent enough training on how to actually do the COWS screening, but then there wasn’t really any information about when to start advocating for meds and things like that.” (nurse focus group, participant 9) • “…I think a lot of the nurses are also talking to each other about how to score COWS, how to do the process of the OUD intervention.” (physician focus group 2, participant 5) • “…it seems like people are actually using COWS more often. When I first started, a lot of people didn’t know where to find COWS or how to score it. Now it seems like enough people are using it reliably that in the mornings when we do rounds, oftentimes our physicians can go into Epic and look at the COWS flow sheet and see what the patient has scored. That is really helpful for our providers so they can prioritize when to see people, and also for the patients, because it is an issue where if a patient gets too uncomfortable, and they know they have a supply of opioids on the street, they will leave, and that’s happened before.” (social worker key informant, participant 2)
Hospital leadership	Limited exposure to messaging and endorsement from system leadership about the OUD intervention	• “I’ve heard crickets from my hospital leadership about this initiative and the OUD intervention.” (nurse key informant, participant 2) • “As a unified healthcare system, hospital leadership should make sure these things get rolled out across all the hospital regions in a way that provides the education that doctors need in order to use the interventions.” (physician key informant, participant 4) • “I feel like there’s a huge gap in education surrounding OUD treatment. I thought there would be an announcement, at least just touched upon, but there hasn’t been any information. I would like to see leadership be on board.” (social worker key informant, participant 4)

**TABLE 4:** Mixed methods display of the quantitative and the qualitative findings.

Topic	Quantitative findings (buprenorphine, methadone, naloxone, and COWS)	Qualitative findings (focus groups, key informant interviews)	Outcomes of data *confirmation, expansion, discordance* and interpretation
Experience with the electronic health record (EHR)-embedded intervention	No significant increase in methadone, naloxone or buprenorphine observed in the post-intervention period.	• Many participants were unaware of the EHR-embedded intervention. • However, participants who used the intervention generally found it to be helpful.	• **Data confirmation:** Lack of awareness of the EHR-embedded intervention may explain the null post-intervention outcomes. • **Data discordance:** When used, the EHR-embedded intervention was a useful tool to initiate methadone and buprenorphine, though this was not reflected in the quantitative outcomes.
Barriers to initiate methadone or buprenorphine	No significant increase in methadone or buprenorphine observed in the post-intervention period.	• Participants reported feeling unprepared to initiate methadone or buprenorphine. • Participants were hesitant to initiate methadone or buprenorphine without system support or without a clear post discharge OUD treatment linkage plan.	• **Data confirmation:** Lack of preparedness to initiate methadone and buprenorphine and uncertainty about post discharge treatment linkage may explain the null post-intervention outcomes. • **Data expansion:** Regular education, access to an addiction specialist, and clear OUD treatment referrals may improve opioid use disorder (OUD) treatment initiation.
Experience with Clinical Opiate Withdrawal Scale (COWS)	Significant increase in COWS documentation observed in the immediate post-intervention period. Change was sustained in the post-intervention period.	• Almost all participants were familiar with COWS, and many reflected on the benefit of using with patients going through withdrawal.	• **Data confirmation:** A mandatory training for COWS was implemented at the system level for nursing staff, which facilitated exposure to COWS and a perceived priority from leadership to include this in patient care • **Data expansion:** COWS was used as a tool to advocate for methadone and buprenorphine initiation and for overall support for patients with OUD going through withdrawal
Hospital leadership	No significant increase in methadone, naloxone, or buprenorphine prescribing observed in the post-intervention period. Significant increase in COWS documentation observed in the immediate post-intervention period with sustained change in the post-intervention period.	• Most participants reported little if any messaging from hospital leadership about the OUD intervention. • Most nurses recalled completing the required online module to complete COWS scoring and documentation.	• **Data confirmation:** Consistent messaging from hospital leadership highlighting the EHR-embedded OUD intervention was lacking, potentially explaining the lack of effect of the intervention on these study outcomes. • **Data confirmation:** Mandated COWS training from nursing leadership demonstrated that this was a priority, potentially explaining the change in COWS scoring in the immediate post-intervention period.
