# Supplementary material for: A mixed methods assessment of an electronic health record-embedded intervention with supportive education and outreach to increase in-hospital opioid use disorder treatment initiation
Source: J Hosp Med. Author manuscript; Available in PMC 2026 Aug 14. (PMC13472743; doi:10.1002/jhm.70289)
Supplement: Supplementary file 1 [file NIHMS2197634-supplement-Appendix_1.docx]

**Appendix 1.** The OUD Working Group, the OUD Educational Components, and the EHR-Embedded OUD Intervention Components

OUD Working Group

Buy-in for interevention development from health system's Chief Medical Officer (CMO) and Chief Nursing Officer (CNO)

CMO-provided dedicated analyst support to build the EHR-embedded intervetion

CNO-approved nurse-required training modules

Addiction medicine physician-hospitalist

Inpatient social work team leader

Inpatient pharmacist and informaticist

Inpatient nursing team leader

OUD Educational and Training Components

Group presentation to each hospital's CMO describing plan to implement an EHR-embedded OUD intervention with educational and training components

On-site presentations (n=5) and live virtual presentations (n=5). Virtural presentations were recorded and available to all health system employees via a shared intranet

Clinician education: Offered to hospitalists physicians and advanced practice professionals

COWS assessment, education and orientation intervetion, methadone and buprenorphine initiation, management of precipitated opioid withdrawal, local OUD treatment referral resources, naloxone prescribing recommendations

Nursing education:

Universal screening (AUDIT-C, DAST-10, CUDIT) and work flow integration

COWS assessment, indications of when to contact clinicians for MOUD initiation based on COWS score, overview of OUD intervention for awareness and comfort

Nursing leadership-required online training modules for COWS assesment

Inpatient social work and pharmacy email communication about OUD intervention

Components of the Non-Interuptive EHR-Embedded Intervention

Pre-specified methadone and buprenorphine orders linked into the EHR medication order prompt

Precipiated opioid withdrawal medication management linked into the EHR medication order prompt

Discharge naloxone orders linked into the discharge medication navigator

Hyperlinks to embedded MDCalc© COWS scoring tool

Hyperlinks to the DSM-5 OUD criteria

Hyperlinks to the National Clinician Consultation Center (NCCC) Substance Use Warmline which offers clinician-to-clinician consultation of substance evaluation and management

Hyperlinks to local substance use treatment programs identified by regional social work leadership

**Appendix 2. ICD 10 codes**

| **ICD 10 codes** | |
| --- | --- |
| **Schizophrenia and other psychotic disorders** | |
| F20.0, F20.1, F20.2, F20.3, F20.5; F20.81, F20.89, F20.9; F25.0, F25.1, F25.8, F25.9; F06.0, F06.2, F22-F24; F29, F32.2, F33.3, F44.89 | |
| **Bipolar disorder** | |
| F30.10-F30.13, F30.2, F30.3, F30.4, F30.8, F30.9, F31.0, F31.10, F31.11, F31.12, F31.13, F31.2, F31.30-F31.32, F31.4, F31.5, F31.60-F31.64, F31.70-F31.78, F31.81, F31.89, F31.9, F33.8, F34.81, F34.89, F34.9, F39 | |
| **Anxiety disorder** | |
| F06.4, F40.00, F40.01, F40.02, F40.10, F40.11, F40.210, F40.218, F40.220, F40.228, F40.230-F40.233, F40.240-F40.243, F40.248, F40.290, F40.291, F40.298, F40.8, F40.9, F41.0, F41.1, F41.3, F41.8, F41.9  F42, F42.2-F42.4, F42.8, F42.9, F43.0-F43.12, F44.9, F45.8, F48.8, F48.9, F93.8, F99, R45.2, R45.5, R45.6, R45.7 | |
| **Depression** | |
| F32.0-F32.5, F32.89, F32.9, F33.0-F33.3, F33.40-F33.42, F33.8, F33.9, F34.1 | |
| **Opioid Poisoning codes** | |
| **Opioid Related Poisoning Codes** | |
| T40.0X1 | [Poisoning by opium, accidental (unintentional)](https://www.aapc.com/codes/icd-10-codes/T40.411) |
| T40.0X2 | [Poisoning by opium, intentional self-harm](https://www.aapc.com/codes/icd-10-codes/T40.412) |
| T40.0X3 | [Poisoning by opium assault](https://www.aapc.com/codes/icd-10-codes/T40.413) |
| T40.0X4 | [Poisoning by opium, undetermined](https://www.aapc.com/codes/icd-10-codes/T40.414) |
| T40.0X5 | [Adverse effect of opium](https://www.aapc.com/codes/icd-10-codes/T40.415) |
| T40.1X1 | [Poisoning by heroin, accidental (unintentional)](https://www.aapc.com/codes/icd-10-codes/T40.411) |
| T40.1X2 | [Poisoning by heroin, intentional self-harm](https://www.aapc.com/codes/icd-10-codes/T40.412) |
| T40.1X3 | [Poisoning by heroin, assault](https://www.aapc.com/codes/icd-10-codes/T40.413) |
| T40.1X4 | [Poisoning by heroin, undetermined](https://www.aapc.com/codes/icd-10-codes/T40.414) |
| T40.1X5 | [Adverse effect of heroin](https://www.aapc.com/codes/icd-10-codes/T40.415) |
| T40.2X1 | Poisoning by other opioids, accidental (unintentional) |
| T40.2X2 | Poisoning by other opioids, intentional self-harm |
| T40.2X3 | Poisoning by other opioids, assault |
| T40.2X4 | Poisoning by other opioids, undetermined |
| T40.2X5 | Adverse effect of other opioids |
| T40.3X1 | Poisoning by other opioids, accidental (unintentional) |
| T40.3X2 | Poisoning by other opioids, intentional self-harm |
| T40.3X3 | Poisoning by other opioids, assault |
| T40.3X4 | Poisoning by other opioids, undetermined |
| T40.3X5 | Adverse effect of other opioids |
| [T40.411](https://www.aapc.com/codes/icd-10-codes/T40.411) | [Poisoning by fentanyl or fentanyl analogs, accidental (unintentional)](https://www.aapc.com/codes/icd-10-codes/T40.411) |
| [T40.41](https://www.aapc.com/codes/icd-10-codes/T40.411)2 | [Poisoning by fentanyl or fentanyl analogs, intentional self-harm](https://www.aapc.com/codes/icd-10-codes/T40.412) |
| [T40.41](https://www.aapc.com/codes/icd-10-codes/T40.411)3 | [Poisoning by fentanyl or fentanyl analogs, assault](https://www.aapc.com/codes/icd-10-codes/T40.413) |
| [T40.414](https://www.aapc.com/codes/icd-10-codes/T40.411) | [Poisoning by fentanyl or fentanyl analogs, undetermined](https://www.aapc.com/codes/icd-10-codes/T40.414) |
| [T40.415](https://www.aapc.com/codes/icd-10-codes/T40.411) | [Adverse effect of fentanyl or fentanyl analogs](https://www.aapc.com/codes/icd-10-codes/T40.415) |
| [T40.421](https://www.aapc.com/codes/icd-10-codes/T40.411) | [Poisoning by tramadol, accidental (unintentional)](https://www.aapc.com/codes/icd-10-codes/T40.411) |
| [T40.42](https://www.aapc.com/codes/icd-10-codes/T40.411)2 | [Poisoning by tramadol, intentional self-harm](https://www.aapc.com/codes/icd-10-codes/T40.412) |
| [T40.42](https://www.aapc.com/codes/icd-10-codes/T40.411)3 | [Poisoning by tramadol, assault](https://www.aapc.com/codes/icd-10-codes/T40.413) |
| [T40.424](https://www.aapc.com/codes/icd-10-codes/T40.411) | [Poisoning by tramadol, undetermined](https://www.aapc.com/codes/icd-10-codes/T40.414) |
| [T40.425](https://www.aapc.com/codes/icd-10-codes/T40.411) | [Adverse effect of](https://www.aapc.com/codes/icd-10-codes/T40.415) tramadol |
| [T40.491](https://www.aapc.com/codes/icd-10-codes/T40.411) | [Poisoning by other synthetic narcotics, accidental (unintentional)](https://www.aapc.com/codes/icd-10-codes/T40.411) |
| [T40.49](https://www.aapc.com/codes/icd-10-codes/T40.411)2 | [Poisoning by other synthetic narcotics, intentional self-harm](https://www.aapc.com/codes/icd-10-codes/T40.412) |
| [T40.49](https://www.aapc.com/codes/icd-10-codes/T40.411)3 | [Poisoning by other synthetic narcotics, assault](https://www.aapc.com/codes/icd-10-codes/T40.413) |
| [T40.494](https://www.aapc.com/codes/icd-10-codes/T40.411) | [Poisoning by other synthetic narcotics, undetermined](https://www.aapc.com/codes/icd-10-codes/T40.414) |
| [T40.495](https://www.aapc.com/codes/icd-10-codes/T40.411) | [Adverse effect of](https://www.aapc.com/codes/icd-10-codes/T40.415) other synthetic narcotics |
| [T40.601](https://www.aapc.com/codes/icd-10-codes/T40.411) | [Poisoning by unspecified narcotics, accidental (unintentional)](https://www.aapc.com/codes/icd-10-codes/T40.411) |
| [T40.602](https://www.aapc.com/codes/icd-10-codes/T40.411) | [Poisoning by unspecified narcotics, intentional self-harm](https://www.aapc.com/codes/icd-10-codes/T40.412) |
| [T40.60](https://www.aapc.com/codes/icd-10-codes/T40.411)3 | [Poisoning by unspecified narcotics, assault](https://www.aapc.com/codes/icd-10-codes/T40.413) |
| [T40.604](https://www.aapc.com/codes/icd-10-codes/T40.411) | [Poisoning by unspecified narcotics, undetermined](https://www.aapc.com/codes/icd-10-codes/T40.414) |
| [T40.605](https://www.aapc.com/codes/icd-10-codes/T40.411) | [Adverse effect of](https://www.aapc.com/codes/icd-10-codes/T40.415) unspecified narcotics |
| T40.691 | [Poisoning by other narcotics, accidental (unintentional)](https://www.aapc.com/codes/icd-10-codes/T40.411) |
| T40.692 | [Poisoning by other narcotics, intentional self-harm](https://www.aapc.com/codes/icd-10-codes/T40.412) |
| T40.693 | [Poisoning by other narcotics, assault](https://www.aapc.com/codes/icd-10-codes/T40.413) |
| T40.694 | [Poisoning by other narcotics, undetermined](https://www.aapc.com/codes/icd-10-codes/T40.414) |
| T40.695 | [Adverse effect of](https://www.aapc.com/codes/icd-10-codes/T40.415) other narcotics |
| **Opioid Use Disorder Codes** | |
| F11.10 | Opioid abuse, uncomplicated |
| F11.12 | Opioid abuse with intoxication |
| F11.13 | Opioid abuse with opioid withdrawal |
| F11.14 | Opioid abuse with opioid induced mood disorder |
| F11.15 | Opioid abuse with opioid induced psychotic disorder |
| F11.18 | Opioid abuse with other opioid induced disorder |
| F11.19 | Opioid abuse with unspecified opioid induced disorder |
| F11.20 | Opioid dependence, uncomplicated |
| F11.22 | Opioid dependence with intoxication |
| F11.23 | Opioid dependence with opioid withdrawal |
| F11.24 | Opioid dependence with opioid induced mood disorder |
| F11.25 | Opioid dependence with opioid induced psychotic disorder |
| F11.28 | Opioid dependence with other opioid induced disorder |
| F11.29 | Opioid dependence with unspecified opioid induced disorder |
| F11.90 | Opioid use, unspecified, uncomplicated |
| F11.92 | Opioid use, unspecified with intoxication |
| F11.93 | Opioid use, unspecified with opioid withdrawal |
| F11.94 | Opioid use, unspecified with opioid induced mood disorder |
| F11.95 | Opioid use, unspecified with opioid induced psychotic disorder |
| F11.98 | Opioid use, unspecified with other opioid induced disorder |
| F11.99 | Opioid use, unspecified with unspecified opioid induced disorder |
| **Psychostimulant Use Disorder Codes** | |
| F15.10 | Amphetamine-type Substance Use Disorder, Mild |
| F15.11 | Amphetamine-type Substance Use Disorder, Mild, in early or sustained remission |
| F15.20 | Amphetamine-type Substance Use Disorder, Moderate |
| F15.21 | Amphetamine-type Substance Use Disorder, Moderate, in early or sustained remission |
| F15.20 | Amphetamine-type Substance Use Disorder, Severe |
| F15.21 | Amphetamine-type Substance Use Disorder, Severe, in early or sustained remission |
| F15.10 | Other or Unspecified Stimulant Use Disorder, Mild |
| F15.11 | Other or Unspecified Stimulant Use Disorder, Mild, In early or sustained remission |
| F15.20 | Other or Unspecified Stimulant Use Disorder, Moderate |
| F15.21 | Other or Unspecified Stimulant Use Disorder, Moderate, In early or sustained remission |
| F15.20 | Other or Unspecified Stimulant Use Disorder, Severe |
| F15.21 | Other or Unspecified Stimulant Use Disorder, Severe, In early or sustained remission |
| **Cocaine Use Disorder codes** | |
| F14.10 | Cocaine Use Disorder, Mild |
| F14.11 | Cocaine Use Disorder, Mild, in early or sustained remission |
| F14.20 | Cocaine Use Disorder, Moderate |
| F14.21 | Cocaine Use Disorder, Moderate, in early or sustained remission |
| F14.20 | Cocaine Use Disorder, Severe |
| F14.21 | Cocaine Use Disorder, Severe, in early or sustained remission |
| **Alcohol Codes** | |
| F10.1X | Alcohol related disorders |
| F10.2X | Alcohol dependence |
| F10.3X-F10.8X | Alcoholic psychosis |
| F10.9X | Alcohol use unspecified |
| K70.0 | Alcoholic fatty liver |
| K70.1 | Alcoholic hepatitis |
| K70.2 | Alcohol fibrosis and sclerosis of liver |
| K70.3 | Alcoholic cirrhosis of liver |
| K70.4 | Alcoholic hepatic failure |
| K70.9 | Alcoholic liver disease, unspecified |
| G62.1 | Alcoholic polyneuropathy |
| G31.2 | Degeneration of nervous system due to alcohol |
| I42.6 | Alcoholic cardiomyopathy |
| K29.2 | Alcoholic gastritis |
| K86.0 | Alcohol induced pancreatitis |
| **Infection Codes** | |
| **Endocarditis** | |
| B376 | Candidal endocarditis |
| I330 | Acute and subacute infective endocarditis |
| I339 | Acute and subacute endocarditis, unspecified |
| I38 | Endocarditis, valve unspecified |
| I39 | Endocarditis and heart valve disorders in diseases classified elsewhere |
| **Skin, Soft-Tissue, and Musculoskeletal Infections** | |
| G062 | Extradural and subdural abscess, unspecified |
| M01X | Direct infection of joint in infectious and parasitic diseases classified elsewhere |
| L02 | Cutaneous abscess, furuncle and carbuncle |
| L03111 | Cellulitis of right axilla |
| L03112 | Cellulitis of left axilla |
| L03113 | Cellulitis of right upper limb |
| L03114 | Cellulitis of left upper limb |
| L03115 | Cellulitis of right lower limb |
| L03116 | Cellulitis of left lower limb |
| L03119 | Cellulitis of unspecified part of limb |
| L03221 | Cellulitis of neck |
| L03222 | Acute lymphangitis of neck |
| M860 | Acute hematogenous osteomyelitis |
| M861 | Other acute osteomyelitis |
| M862 | Subacute osteomyelitis |
| M868 | Other osteomyelitis |
| M000 | Staphylococcal arthritis and polyarthritis |
| M009 | Pyogenic arthritis, unspecified |
| B958 | Unspecified staphylococcus as the cause of diseases classified elsewhere |

| **Appendix 3.** Results of the ITS Analysis Demonstrating the Percentage of Hospitalizations with an Outcome of Interest (Percentage of Monthly Administrations of Methadone, Buprenorphine Discharge Prescriptions, Naloxone Discharge Prescriptions, COWS Documentation) among Patients with Documented OUD and/or Opioid Poisoning Pre- and Post-Implementation of the EHR-Embedded OUD Intervention by Region (North, Central, or South)^1^ | | | | |
| --- | --- | --- | --- | --- |
| **Model Outcome and Region** | **Variable** | **Estimate** | **Standard Error** | **P-Value** |
| **≥30mg Methadone Administration** | | | | |
| North Region | Slope Pre-Intervention Period | 0.22 | 0.062 | 0.001 |
|  | Slope Post-Intervention Change | -0.07 | 0.115 | 0.547 |
|  | Level change | -2.804 | 1.44 | 0.06 |
| Central Region | Slope Pre-Intervention Period | 0.284 | 0.099 | 0.007 |
|  | Slope Post-Intervention Change | 0.104 | 0.182 | 0.570 |
|  | Level change | -3.453 | 2.289 | 0.14 |
| South Region | Slope Pre-Intervention Period | 0.015 | 0.095 | 0.876 |
|  | Slope Post-Intervention Change | -0.019 | 0.174 | 0.913 |
|  | Level change | -0.769 | 2.189 | 0.727 |
| **Discharge Buprenorphine Prescription** | | | | |
| North Region | Slope Pre-Intervention Period | 0.117 | 0.072 | 0.114 |
|  | Slope Post-Intervention Change | -0.282 | 0.133 | 0.040^2^ |
|  | Level change | 3.059 | 1.675 | 0.076 |
| Central Region | Slope Pre-Intervention Period | 0.258 | 0.071 | 0.001 |
|  | Slope Post-Intervention Change | -0.366 | 0.135 | 0.01^2^ |
|  | Level change | -0.524 | 1.661 | 0.754 |
| South Region | Slope Pre-Intervention Period | 0.252 | 0.064 | 0.001 |
|  | Slope Post-Intervention Change | -0.355 | 0.1205 | 0.006^2^ |
|  | Level change | -1.390 | 1.4746 | 0.352 |
| **Discharge Naloxone Prescription** | | | | |
| North Region | Slope Pre-Intervention Period | -0.048 | 0.092 | 0.609 |
|  | Slope Post-Intervention Change | 0.034 | 0.169 | 0.840 |
|  | Level change | 3.001 | 2.131 | 0.167 |
| Central Region | Slope Pre-Intervention Period | 0.331 | 0.105 | 0.003 |
|  | Slope Post-Intervention Change | 0.103 | 0.193 | 0.599 |
|  | Level change | -0.497 | 2.434 | 0.839 |
| South Region | Slope Pre-Intervention Period | 0.293 | 0.113 | 0.0133 |
|  | Slope Post-Intervention Change | -0.042 | 0.207 | 0.8392 |
|  | Level change | -4.007 | 2.609 | 0.133 |
| **COWS Score** | | | | |
| North Region | Slope Pre-Intervention Period | 0.06 | 0.023 | 0.013 |
|  | Slope Post-Intervention Change | -0.233 | 0.043 | <.001^2^ |
|  | Level change | 7.065 | 0.536 | <.001^3^ |
| Central Region | Slope Pre-Intervention Period | 0.230 | 0.104 | 0.034 |
|  | Slope Post-Intervention Change | -0.550 | 0.192 | 0.007^2^ |
|  | Level change | 11.414 | 2.417 | <.001^3^ |
| South Region | Slope Pre-Intervention Period | -0.098 | 0.065 | 0.142 |
|  | Slope Post-Intervention Change | 0.609 | 0.119 | <.001^2^ |
|  | Level change | 2.335 | 1.505 | 0.129 |

^1^ The three northern hospitals included a 263-bed hospital, a 191-bed hospital, and a 91-bed hospital, the five central hospitals included a 775-bed hospital (which has an addiction consultation services Monday through Saturday), a 93-bed hospital, 83-bed hospital, and a 34-bed hospital, and the four southern hospitals included a 387-bed hospital, a 140-bed hospital, a 13-bed hospital, and a 22-bed hospital.

^2^ Significant changes in the post intervention period compared to the pre-intervention period.

^3^ Significant changes in the immediate post intervention period, i.e., level change.

**Appendix 4.** Results of the ITS Analysis Demonstrating the Percentage of Hospitalizations with an Outcome of Interest (Percentage of Monthly Administrations of Methadone, Buprenorphine Discharge Prescriptions, Naloxone Discharge Prescriptions, COWS Documentation) among Patients with Documented OUD and/or Opioid Poisoning Pre- and Post-Implementation of the EHR-Embedded OUD Intervention by Region (North, Central, or South)^1^

**≥30 mg Methadone Administration**

**North**

**Central**

**South**

**Buprenorphine Discharge Prescriptions**

**North**

**Central**

**South**

**Naloxone Discharge Prescriptions**

**North**

**Central**

**South**

**North**

**Central**

**South**

**COWS Score**

^1^ The three northern hospitals included a 263-bed hospital, a 191-bed hospital, and a 91-bed hospital. The five central hospitals included a 775-bed hospital (which has an addiction consultation services Monday through Saturday), a 93-bed hospital, 83-bed hospital, and a 34-bed hospital. The four southern hospitals included a 387-bed hospital, a 140-bed hospital, a 13-bed hospital, and a 22-bed hospital
